# Cardiac T1 mapping enables risk prediction of LV dysfunction after surgery for aortic regurgitation

**DOI:** 10.3389/fcvm.2023.1155787

**Published:** 2023-06-23

**Authors:** Martin Sinn, Johannes Petersen, Alexander Lenz, Maria von Stumm, Tatiana Maria Sequeira Groß, Lukas Huber, Hermann Reichenspurner, Gerhard Adam, Gunnar Lund, Peter Bannas, Evaldas Girdauskas

**Affiliations:** ^1^ Department of Diagnostic and Interventional Radiology, University Hospital Eppendorf, Hamburg, Germany; ^2^ Department of Cardiovascular Surgery, University Hospital Eppendorf, Hamburg, Germany; ^3^ Department of Cardiothoracic Surgery, Augsburg University Hospital, Augsburg, Germany

**Keywords:** aortic valva insufficiency, cardiac MRI, repair, replacement, t1 mapping

## Abstract

**Background:**

To assess whether cardiac T1 mapping for detecting myocardial fibrosis enables preoperative identification of patients at risk for early left ventricular dysfunction after surgery of aortic regurgitation.

**Methods:**

1.5 Tesla cardiac magnetic resonance imaging was performed in 40 consecutive aortic regurgitation patients before aortic valve surgery. Native and post-contrast T1 mapping was performed using a modified Look-Locker inversion-recovery sequence. Serial echocardiography was performed at baseline and 8 ± 5 days after aortic valve surgery to quantify LV dysfunction. Receiver operating characteristic analysis was performed to determine the diagnostic accuracy of native T1 mapping and extracellular volume for predicting postoperative LV ejection fraction decrease >−10% after aortic valve surgery.

**Results:**

Native T1 was significantly increased in patients with a postoperatively decreased LVEF (*n* = 15) vs. patients with a preserved postoperative LV ejection fraction (*n* = 25) (i.e., 1,071 ± 67 ms vs. 1,019 ± 33 ms, *p* = .001). Extracellular volume was not significantly different between patients with preserved vs. decreased postoperative LV ejection fraction. With a cutoff-of value of 1,053 ms, native T1 yielded an area under the curve (AUC) of .820 (95% CI: .683–.958) for differentiating between patients with preserved vs. reduced LV ejection fraction with 70% sensitivity and 84% specificity.

**Conclusion:**

Increased preoperative native T1 is associated with a significantly higher risk of systolic LV dysfunction early after aortic valve surgery in aortic regurgitation patients. Native T1 could be a promising tool to optimize the timing of aortic valve surgery in patients with aortic regurgitation to prevent early postoperative LV dysfunction.

## 1. Introduction

Aortic regurgitation (AR) is a common heart valve disease in Western countries, and its incidence increases with patient age (1). Chronic aortic regurgitation remains compensated for a long time and becomes symptomatic when progressive left heart failure develops. Currently, the therapeutic strategy in asymptomatic AR patients without signs of impaired LV function is watchful waiting, while symptomatic patients are treated by aortic valve surgery (2). However, left ventricular (LV) dysfunction can persist even after successful aortic valve surgery for AR, and the precursor is early postoperative systolic dysfunction. Furthermore, a postoperative LVEF decline and persistence of reduced LVEF are associated with significantly higher long-term mortality after aortic valve surgery in patients with aortic regurgitation (3). Therefore, identifying AR patients at risk for systolic LV dysfunction after aortic valve surgery has apparent clinical relevance.

Adequate timing of valvular intervention is one of the key factors determining patients' outcome in valvular heart disease (3). If aortic valve surgery is performed too late, patients may suffer from ongoing postoperative LV dysfunction after valvular intervention due to progressive myocardial fibrosis (4, 5). The timing of surgery is complex and depends on a combination of clinical and echocardiographic characteristics. Myocardial tissue analysis by cardiac magnetic resonance imaging (CMR) currently has no impact on surgical decision-making regarding the timing of intervention. However, current CMR mapping techniques show a strong correlation between native T1 and histologic collagen volume fraction, determining the degree of myocardial fibrosis (6). In addition, a previous consensus statement underlined the value of native T1 for visualization and quantification of diffuse myocardial fibrosis (7). However, it is important to take note, that high native T1 values also can indicate the presence of myocardial edema, hyperemia, and capillary leak (8).

We hypothesized that diffuse interstitial myocardial fibrosis might be present in AR patients who experience early systolic LV dysfunction after aortic valve surgery. Therefore, we aimed to evaluate the association between preoperative cardiac T1 mapping for detecting myocardial fibrosis and early systolic LV dysfunction after surgery for aortic regurgitation.

## 2. Material and methods

### 2.1. Subjects

The institutional ethics committee approved this prospective study, and all subjects gave written informed consent. M.S, J.P., P.B. and E.G. had full control of the data and the material submitted for publication.

Consecutive patients with severe aortic regurgitation referred for aortic valve surgery between July 2016 and August 2019 were prospectively enrolled in the study. Study exclusion criteria were as follows: history of coronary artery disease, acute aortic valve disease (i.e., type A aortic dissection or infectious endocarditis), and common contraindications for MRI such as severe obesity and metallic foreign bodies.

The severity of aortic regurgitation was defined by established diagnostic criteria, including transthoracic echocardiography and clinical aspects (2).

### 2.2. Procedures and techniques

All patients underwent cardiac MR imaging (3 ± 5 days) before aortic valve surgery, which was conducted in an identical fashion in all patients using a 1.5 Tesla MR imaging unit (Achieva; Philips Medical Systems, Best, the Netherlands). The protocol included standard steady-state free-precession cine MR. Inversion-recovery LGE MR imaging was performed after intravenous administration of 0.075 mmol gadobenate dimeglumine (MultiHance; Bracco, Konstanz, Germany). Native (non-contrast) and post-contrast T1 mapping were performed using a 5s(3s)3s modified Look-Locker inversion-recovery (MOLLI) sequence on three short-axis sections (apical, middle, and basal) before and 15 min after contrast agent administration (9, 10). Two radiologists (M.S. and L.H., six and two years of experience in reading CMR images) independently and blindly analyzed each MR set in random order. Corresponding native T1, post-contrast T1, and extracellular volume (ECV) maps were generated using CVI42 (Circle Cardiovascular Imaging Inc., Calgary, AB, Canada) by using the following previously established equation: ECV = *λ* *(1−hematocrit) with *λ* = ΔR1myocardium/ΔR1blood (where R1 = 1/T1) (11). Hematocrit was measured from a venous blood sample obtained on the same day of the MR imaging examination. Global mapping values were measured by drawing a single ROI in the septum on mid-cavity short-axis maps (7). Special attention was paid not to include the blood volume or epicardial fat in the measurements. Parametric CMR parameters are given as the mean of the two observers.

Preoperative echocardiography was performed at hospital admission (i.e., one day before surgery). Before hospital discharge, echocardiography was repeated by the same investigators and using the same echocardiographic criteria after the mean of 8 ± 5 days postoperatively. All echocardiographic examinations were meticulously conducted by senior cardiologists with substantial expertise in echocardiography. Each examination was performed by one cardiologist under the careful supervision of another using a validation process based on the four-eye principle.

Aortic valve surgery was performed via partial upper mini-sternotomy, using a standard cardiopulmonary bypass and mild systemic hypothermia in all patients. The primary surgical strategy was aortic valve repair whenever possible. The remaining patients underwent mechanical/tissue aortic valve replacement. The patient's characteristics were gathered retrospectively from their medical record at the hospital, while the intraoperative characteristics were documented based on the surgical note.

Systolic LV dysfunction after aortic valve surgery was defined by the difference between left ventricular ejection fraction (LVEF) in the pre- and postoperative echocardiography. Patients with an LVEF that improved after surgery, stayed equal, or decreased by less than −10% were considered as patients with a preserved LVEF. Conversely, patients with an LVEF decrease by −10% or more were considered as patients with a postoperative systolic LV dysfunction.

### 2.3. Statistical tests

Statistical analysis was performed using SPSS for Mac OS X, version 26.0 (IBM SPSS Statistics, Armonk, NY, USA). Graphs were plotted using Prism 7.0 for Mac OS X (GraphPad Software Inc., San Diego, CA, USA).

Continuous data are given as mean ± SD, and categorical data are presented as absolute numbers and percentages. Normal distribution was tested using the D'Agostino-Pearson test. *P* values were calculated by the Mann-Whitney U test, paired Wilcoxon test, *χ*^2^ test, or Fisher's exact test, as appropriate. Interobserver agreement was determined by using the intraclass correlation coefficient.

Receiver operating curve (ROC)-analysis of preoperative parametric CMR mapping was performed in patients with aortic regurgitation for differentiating between patients with preserved and decreased LVED after aortic valve surgery. We calculated the areas under the ROC curves (AUCs), and the optimal cutoff values from the ROC curves were identified using the Youden index. *P* < 0.05 was considered to indicate a statistically significant difference.

## 3. Results

### 3.1. Baseline study population

**Figure 1:**
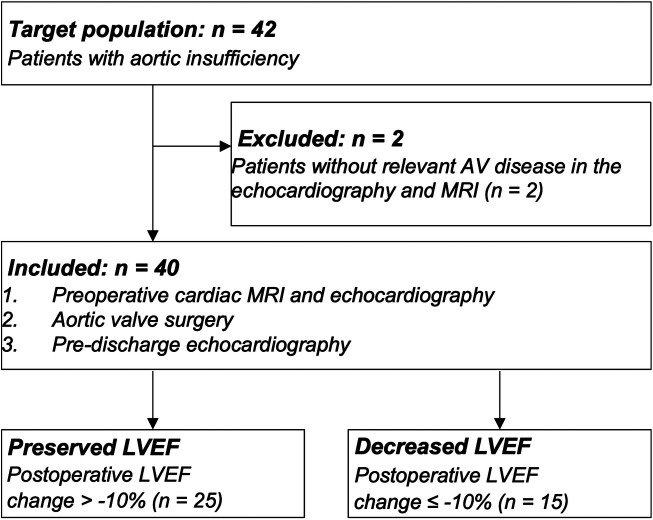
Flow diagram of inclusion and exclusion criteria. Patients with aortic valve disease that underwent preoperative MRI, echocardiography, and follow-up echocardiography after aortic valve surgery were included. Patients were separated into two groups after surgery based on the comparison of echocardiography-derived preoperative and postoperative left ventricular ejection fractions (LVEF): LVEF was defined as preserved if the change were <−10% and as decreased if the changes were ≥−10%.

A total of 40 consecutive AR patients referred for aortic valve surgery were included (Figure 1). Preoperative cardiac MRI and pre- and postoperative echocardiography parameters in the whole study cohort are summarized in Table 1. Briefly, our study cohort consisted of relatively young (i.e., mean age 51 ± 14 years) and predominantly male patients [i.e., 34 (85%) men]. Aortic valve morphotype was almost equally distributed tricuspid vs. congenital (i.e., bicuspid aortic valve/unicuspid aortic valve; BAV/UAV) aortic valve disease. Two-thirds of the study cohort underwent aortic valve repair, while the remaining had aortic valve replacement (*n* = 14, 35%).

**Table 1:** Characteristics of patients with aortic regurgitation (*n* = 40).

Aortic regurgitation patients	All patients	Preserved EF	Decreased EF	*P*
	*n* = 40	*n* = 25	*n* = 15	Preserved vs. decreased EF
Age at time of operation (y)	51 ± 14	52 ± 14	49 ± 13	.513
Gender, male	34 (85)	21 (84)	13 (87)	.600
Aortic valve morphotype				
TAV	22 (55)	16 (64)	6 (40)	.336
BAV/UAV	18 (45)	9 (36)	9 (60)	.336
Baseline echocardiography				
Ejection fraction (%)	56 ± 9	53 ± 11	59 ± 5	.514
LVEDD (mm)	64 ± 7	63 ± 7	65 ± 8	.795
Grade of regurgitation				.819
None or mild	0 (0)	0 (0)	0 (0)	
Moderate	6 (15)	4 (16)	2 (13)	
Severe	34 (85)	21 (84)	13 (86)	
Baseline CMR characteristics				
LVEDV (ml)	231 ± 90	254 ± 81	284 ± 90	.650
LVESV (ml)	102 ± 56	116 ± 58	129 ± 49	.262
LVSV (ml)	130 ± 43	135 ± 42	158 ± 44	.074
Ejection fraction (%)	58 ± 11	55 ± 14	56 ± 5	.128
Native T1 (ms)	1,039 ± 54	1,019 ± 33	1,071 ± 67	.001
ECV (%)	28 ± 2	29 ± 2	28 ± 3	.466
Surgical procedure				
Aortic valve repair	26 (65)	14 (56)	12 (80)	.177
Aortic valve replacement	14 (35)	11 (44)	3 (20)	.177
Aortic clamp time (min)	80 ± 31	87 ± 32	70 ± 27	.117
Cardiopulmonary bypass time (min)	126 ± 40	133 ± 43	115 ± 35	.202
Postoperative peak blood parameters				
Creatine kinase (U/L)	1,090 ± 2,080	741 ± 419	1,691 ± 3,375	.144
Creatine kinase MB (%)	6.7 ± 12.2	5.8 ± 4.6	7.3 ± 12.1	.889
Troponin T (pg/ml)	539 ± 433	609 ± 502	422 ± 287	.428
Postoperative echocardiography				
Ejection fraction (%)	48 ± 10	52 ± 10	42 ± 6	.001
LVEDD (mm)	54 ± 6	54 ± 7	56 ± 6	.438
Grade of regurgitation				.256
None	31 (78)	21 (84)	10 (67)	
Mild	9 (23)	4 (16)	5 (33)	
Moderate or severe	0 (0)	0 (0)	0 (0)	

Data are mean ± SD or *n* (%).

*BAV*, bicuspid aortic (Sievers type 1) valve with fusion of the left and right cusps (L/R); *UAV*, unicuspid (unicommissural) aortic valve morphotype; *TAV*, tricuspid aortic valve.

### 3.2. Postoperative vs. preoperative LVEF change

**Figure 2:**
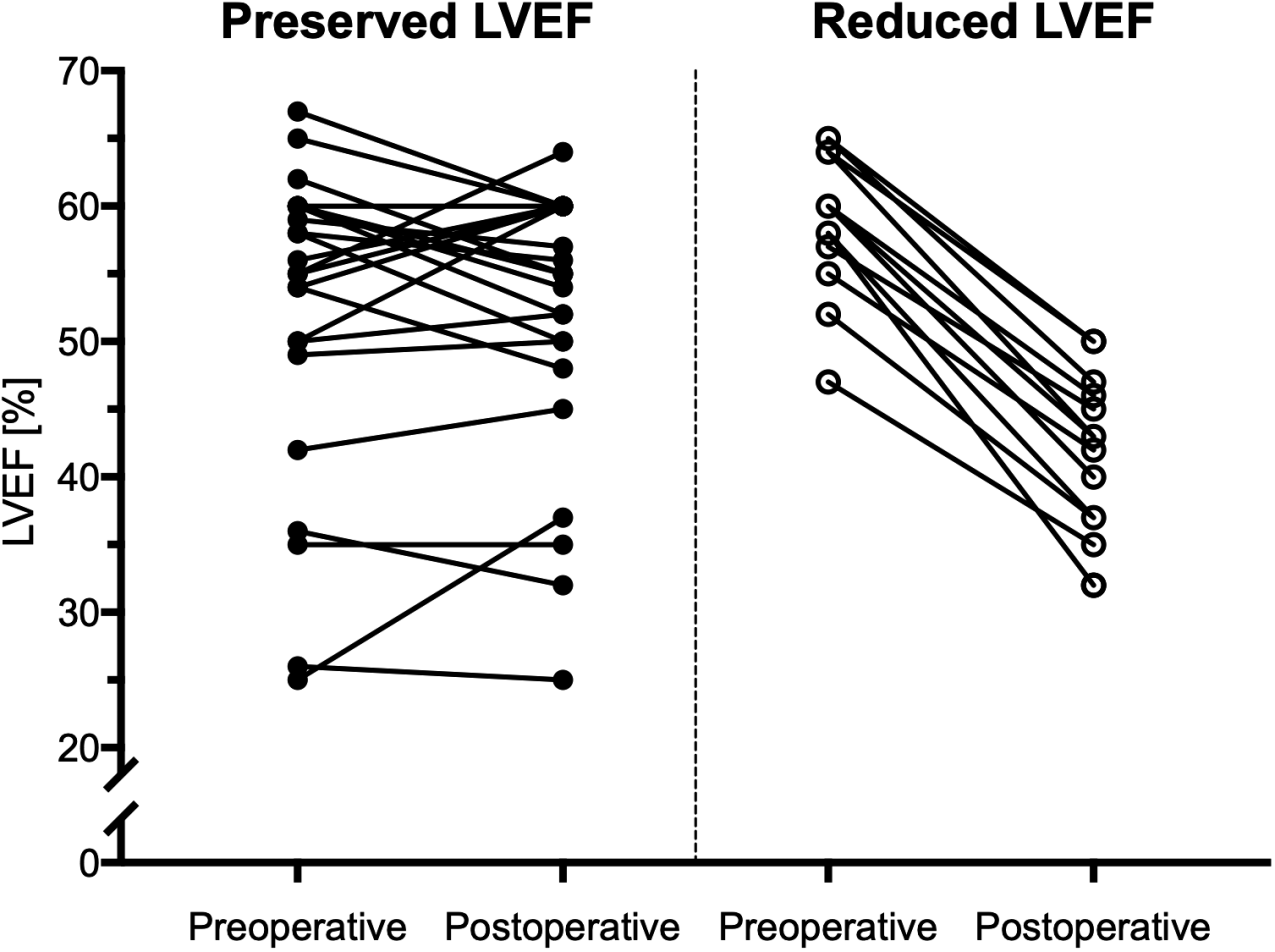
Course of the LVEF in patients with preserved and reduced LVEF. Twenty-five patients with aortic regurgitation showed a preserved postoperative LVEF with a postoperative change of less than −10%. On the other hand, fifteen patients developed a reduced postoperative LVEF, which declined by more than −10%.

Our study cohort was separated into two groups, according to the echocardiographic change in the postoperative vs. preoperative LVEF: 25 patients (63%) had a postoperatively preserved LVEF, while the 15 remaining patients (37%) had a decreased LVEF. In the subgroup of patients with a postoperatively preserved LVEF, mean preoperative LVEF was 53 ± 11% and remained stable after the surgery with 52 ± 10% (*p* = .855). However, in the subgroup of patients with postoperative decreased LVEF, mean preoperative LVEF was 59 ± 5% and decreased postoperatively significantly to 42 ± 6% (*p* < .001) (Figure 2).

**Figure 3:**
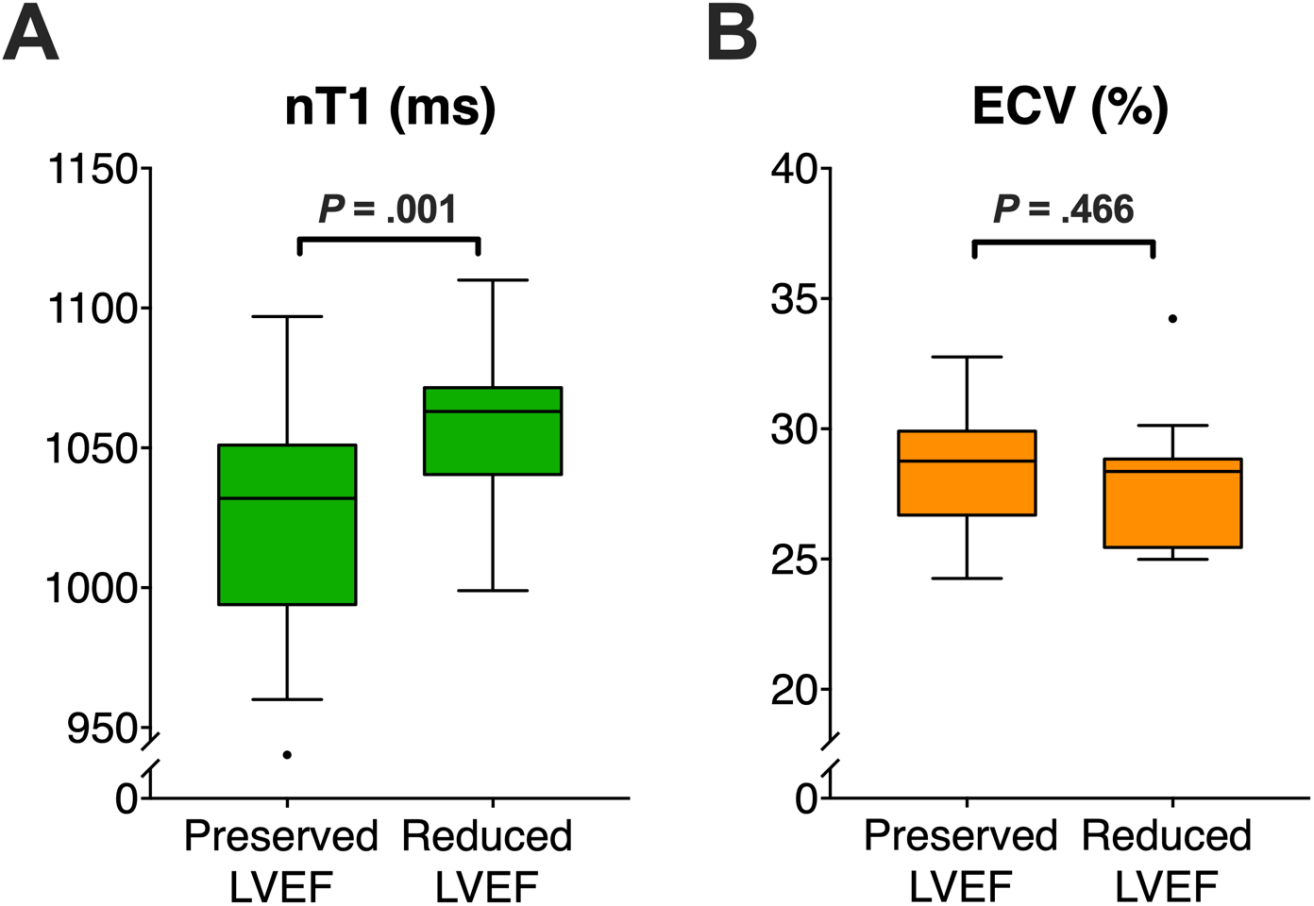
Comparison of preoperative parametric MR maps between patients with preserved and decreased LVEF after aortic valve surgery. In a 69-year-old TAV patient with post-operatively preserved LVEF (+2% change), preoperative native T1 and ECV showed normal values (1,003 ms and 26%). In contrast, in a 49-year-old BAV patient with post-operatively decreased LVEF (−15% change), native T1-map was prolonged (1,112 ms) and ECV-map was increased (30%). The black-outlined polygon illustrates a representative region of interest (ROI), demonstrating how parametric values were obtained. *ECV*, extracellular volume, *LVEF*, left ventricular ejection fraction.

### 3.3. Preoperative clinical and LV function parameters

There were no significant differences in the demographic variables between the cohorts with preserved vs. decreased LVEF (Table 1). In a detailed analysis, neither the cardiopulmonary bypass time nor the aortic clamp time exhibited statistically significant differences between the two cohorts, with *p*-values of .202 and .117 respectively. Furthermore, the preoperative echocardiographic and MR parameters of LV size/function were comparable between both study subgroups (Table 1). Similarly, there was no difference in surgical procedures performed between patients with preserved vs. decreased LVEF (Table 1). Correlation between LVEF assessed by CMR and echocardiography before surgery showed an ICC of .759. The postoperative markers of myocardial injury, such as peak creatine kinase, peak creatine kinase MB, or peak troponin T, were comparable in both groups and therefore failed to explain the decline of LV function in patients with a postoperatively reduced LVEF (Table 1).

### 3.4. Preoperative LGE and mapping CMR parameters

In total, eight patients (20%) showed a non-ischemic LGE, thereof three patients (12%) had a postoperatively preserved EF, and five (33%) had a decreased EF (*p* = 126). None of the patients showed an ischemic LGE.

**Figure 4:**
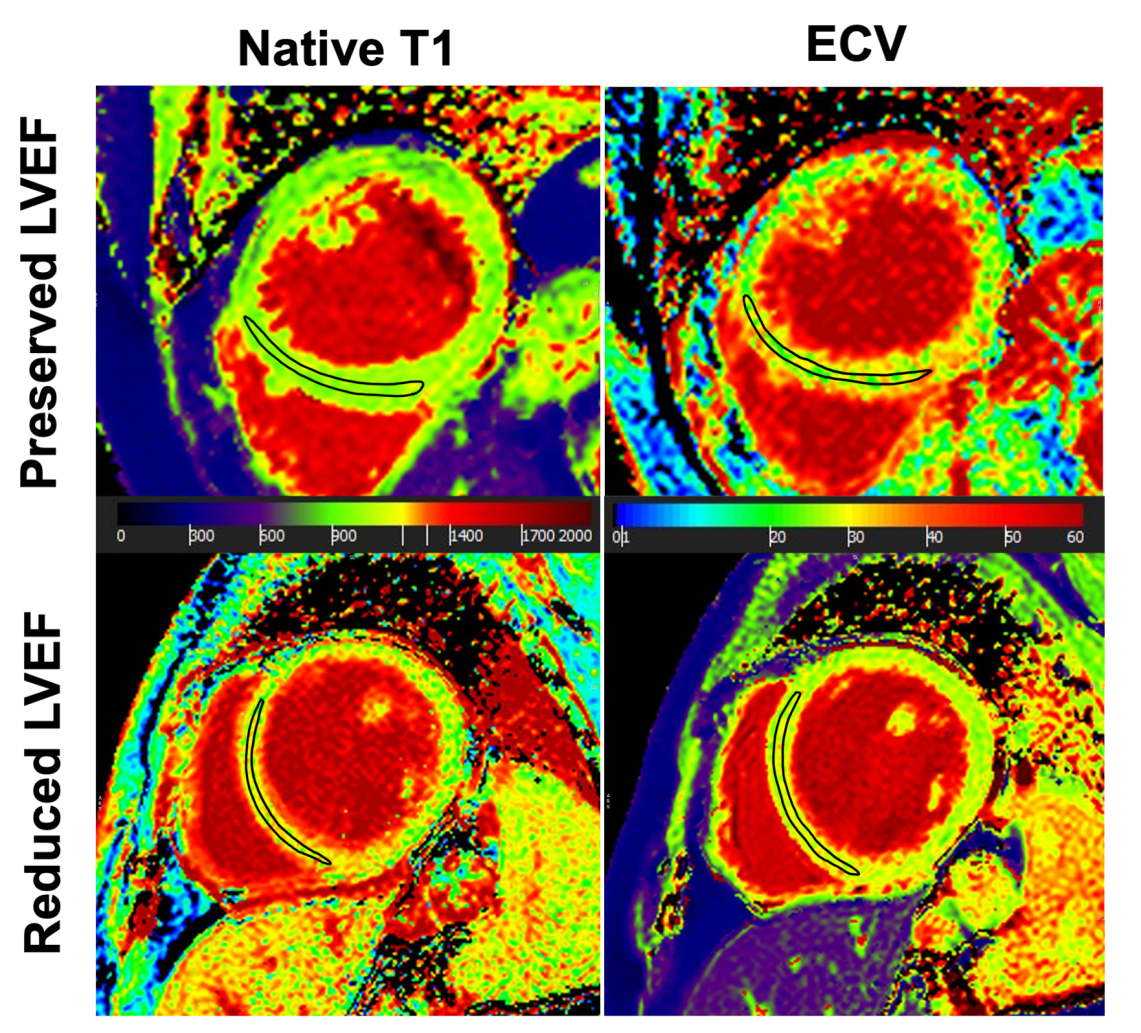
Comparison of preoperative parametric MR mapping in 40 patients with aortic regurgitation. (**A**) Preoperative native T1-times are significantly higher in regurgitation patients with post-operatively decreased LVEF vs. patients with post-operatively preserved LVEF. (**B**) Preoperative ECV does not significantly differ between patients with decreased vs. preserved LVEF. *LVEF*, left ventricular ejection fraction, *ECV*, extracellular volume.

The subgroup of patients with a postoperatively decreased LVEF had significantly longer preoperative native T1 times as compared to the subgroup with a preserved postoperative LVEF (i.e., 1,071 ± 67 ms vs. 1,019 ± 33 ms, *p* = .001), Figure 3). As opposite to this finding, ECV values were comparable between patients with decreased vs. preserved LVEF with (i.e., 29 ± 2% vs. 28 ± 3%, *p *= .466) (Figure 3). ICC for measuring native T1 and EVC revealed excellent agreement between the two observers with .876, and .869, respectively, indicating a high reproducibility. Figure 4 presents the parametric MR maps of two representative patients, one with preserved and one with decreased LVEF after aortic valve surgery.

### 3.5. ROC analysis

**Figure 5:**
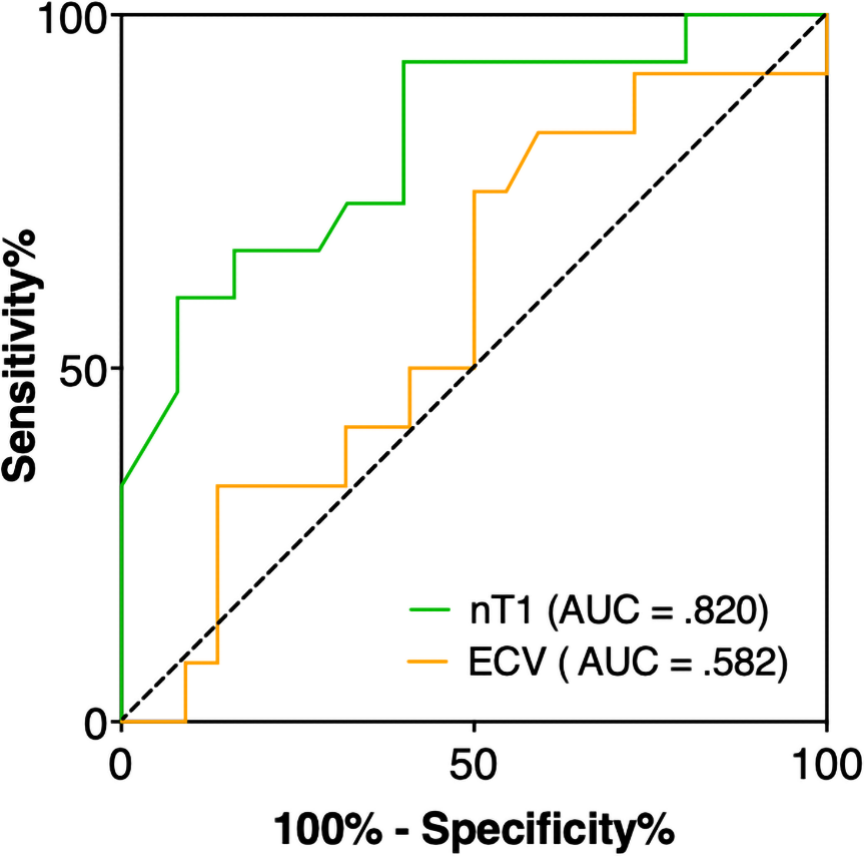
ROC analysis revealed that native T1 had the best performance with an AUC of 0.820 (95% CI: 0.683–0.958) to identify aortic regurgitation patients with early decreased LVEF after aortic valve surgery, whereas ECV had a lower AUC of 0.582 (95% CI: 0.380–0.783). A T1 time ≥1,053 ms achieved a sensitivity of 70% and specificity of 84% to differentiate between patients with preserved and decreased LVEF.

Native T1 values yielded the best area under the ROC curve (AUC) of .820 (95% CI: .683–.958) for differentiation between postoperatively preserved vs. decreased LVEF in AR patients (Figure 5). Cut-off value of T1 ≥1,053 ms showed good sensitivity of 70% and high specificity of 84% to identify those patients with postoperative systolic LV dysfunction. On the other hand, ECV had lower AUC with 0.582 (95% CI: 0.380–0.783) respectively.

## 4. Discussion

Our study aimed to analyze the association between preoperative T1 mapping values defined by CMR and decreased LVEF after aortic valve surgery for aortic regurgitation. Our data revealed that patients with postoperatively decreased LVEF had significantly higher preoperative native T1 values than those with postoperatively preserved LVEF. Subsequent ROC analysis showed that native T1 of >1,053 ms was the optimal cutoff value to predict postoperative systolic LV dysfunction with a sensitivity of 70% and specificity of 84%.

Identifying those patients at risk for systolic LV dysfunction after aortic valve surgery is a highly relevant clinical issue. Markedly reduced LVEF is associated with a significantly reduced ten years survival of 56 ± 5% after aortic valve replacement compared to 70 ± 3% survival in patients with preoperatively normal LVEF (12). Petersen et al. identified that a persistence of reduced LVEF is associated with reduced long-term survival after aortic valve surgery in AR patients (3).

The presence of myocardial fibrosis has been shown to impact the postoperative outcome after aortic valve surgery regarding systolic LV function and survival. Some previous data revealed that patients with severe AR also had pronounced myocardial fibrosis at the time of valve surgery (4, 5, 13). Furthermore, the amount of fibrosis was inversely correlated with LV functional improvement and the long-term survival alter valvular surgery in patients with severe AR. Such findings underline the negative effect of myocardial fibrosis on LV function and survival (14).

More recent CMR studies proposed native T1 and ECV as reliable parameters to quantify diffuse interstitial fibrosis and showed a good correlation between native T1/ECV and histological collagen fraction (6, 15, 16). In addition, another MR study showed that native T1 and ECV have good diagnostic accuracies in identifying diffuse myocardial fibrosis in patients with valvular heart disease, including AR (17).

Our study confirms the value of T1 mapping for detecting silent myocardial dysfunction in chronic severe aortic regurgitation patients. We analyzed preoperative native T1 and ECV values to correlate them with the postoperative decline of LV function after surgery for AR. We found that native T1 had the best association with the postoperative LVEF decline of ≥10%. Furthermore, native T1 had significantly higher AUC than ECV values, indicating that ECV is inferior to identifying the postoperative LVEF decrease. Of note, ECV values only indicate the increase of the extracellular space. In contrast, native T1 additionally correlates with myocardial edema, hyperemia, and capillary leak (8), and these factors may play a role in the postoperative LVEF decline. This could suggest that factors beyond fibrosis, such as myocardial edema, hyperemia, or capillary leak, could have had a significant impact on postoperative LVEF in our cohort. It is also possible that the interplay between these factors is more complex and thus better captured by native T1 mapping, which may explain the observed differences in performance between the two techniques.

Over one-third of our study cohort had an early postoperative LVEF decline of >10%. Interestingly, almost all patients with a postoperative decline of LV function had a normal preoperative LVEF (i.e., mean of 59% ± 5%) (Figure 2). This finding is in accordance with the previous studies demonstrating that AR patients are a high-risk population for increased perioperative morbidity and mortality. For example, a previous study showed that AR patients had significantly lower LVEF recovery rates after surgery as compared to aortic stenosis (AS) patients who had LVEF recovery rate (i.e., 0.7 percent points/year for AR vs. 2.8 percent points/year for AS, *p* < 0.01) (18). Furthermore, the rate of adverse cardiac events was significantly higher in AR patients vs. AS patients (18).

Of note, we observed no differences in the postoperative markers of myocardial ischemic injury, including peak creatine kinase MB and peak troponin T between both study groups, which might have potentially explained the postoperative LVEF decline. Therefore, other factors must be responsible for the early postoperative LVEF decline, as observed in more than one-third of our AR patients. The exact mechanisms responsible for the early postoperative LVEF decline are currently unknown and should be the focus of subsequent studies.

### 4.1. Clinical implications

Our study has some important clinical implications and serves as a background for a further prospective trial. We hypothesize that native T1 mapping could be a promising imaging marker to predict systolic LV dysfunction after aortic valve surgery in patients with aortic regurgitation and may, therefore, guide the timing of surgical intervention. Thus, native T1 values might be used to indicate aortic valve surgery in the future, e.g., based on the cutoff value >1,053 ms. Furthermore, the high specificity value of native T1 is beneficial to include patients at high risk for systolic LV dysfunction after aortic valve surgery with a high probability and low rate of false positives. Future studies have to show that operating at average or slightly increased T1 values reduces the number of patients with early LV dysfunction and that there is a cutoff, which prevents the occurrence of LV dysfunction at all, as a proof of concept.

### 4.2. Limitations

While our findings hold significant promise, it's crucial to acknowledge the limitations of this study, most notably our relatively small sample size. We fully recognize that the generalizability of our results may be limited by this factor. As such, it is vital to exercise caution when interpreting these findings. To solidify the value and applicability of our results, larger, prospective validation studies are indeed necessary. It is through these expanded inquiries that we can truly substantiate and build upon our preliminary insights.

Echocardiography examinations were performed by experienced senior cardiologists based on the four-eye principle. However, we must clarify that each echocardiographic assessment was performed individually and not duplicated, precluding the calculation of interobserver agreement. This single-operator nature of the measurements may present a limitation in terms of assessing the reproducibility of our results.

### 4.3. Summary, conclusion, and future directions

Preoperative cardiac T1 mapping has good sensitivity and high specificity values to identify those patients at increased risk for systolic LV dysfunction early after aortic valve surgery for AR. Therefore, implementing T1 mapping in patients with chronic severe AR as an imaging biomarker may guide the timing of aortic valve intervention in the future and will be the focus of ongoing research.

## Data Availability

The raw data supporting the conclusions of this article will be made available by the authors, without undue reservation.
